# Ferroptosis and Autophagy-Related Genes in the Pathogenesis of Ischemic Cardiomyopathy

**DOI:** 10.3389/fcvm.2022.906753

**Published:** 2022-06-30

**Authors:** Yue Zheng, Wenqing Gao, Qiang Zhang, Xian Cheng, Yanwu Liu, Zhenchang Qi, Tong Li

**Affiliations:** ^1^School of Medicine, Nankai University, Tianjin, China; ^2^Department of Heart Center, The Third Central Hospital of Tianjin, Tianjin, China; ^3^Nankai University Affiliated Third Center Hospital, Nankai University, Tianjin, China; ^4^Tianjin Key Laboratory of Extracorporeal Life Support for Critical Diseases, Tianjin, China; ^5^Artificial Cell Engineering Technology Research Center, Tianjin, China; ^6^Department of Heart Center, The Third Central Clinical College of Tianjin Medical University, Tianjin, China

**Keywords:** GEO, ferroptosis, autophagy, myocardial infarction, progression, IL-6, single cell sequencing, pathway enrichment analysis

## Abstract

**Background:**

Obesity plays an important role in type 2 diabetes mellitus (T2DM) and myocardial infarction (MI). Ferroptosis and ferritinophagy are related to metabolic pathways, such as fatty acid metabolism and mitochondrial respiration. We aimed to investigate the ferroptosis- and autophagy-related differentially expressed genes (DEGs) that might be potential targets for MI progression.

**Methods:**

GSE116250 was analyzed to obtain DEGs. A Venn diagram was used to obtain the overlapping ferroptosis- and autophagy-related DEGs. The enrichment pathway analysis was performed and the hub genes were obtained. Pivotal miRNAs, transcription factors, and drugs with the hub genes interactions were also predicted. The MI mice model was constructed, and qPCR analysis and single-cell sequencing were used to validate the hub genes.

**Results:**

Utilizing the limma package and the Venn diagram, 26 ferroptosis-related and 29 autophagy-related DEGs were obtained. The list of ferroptosis-related DEGs was analyzed, which were involved in the cellular response to a toxic substance, cellular oxidant detoxification, and the IL-17 signaling pathway. The list of autophagy-related DEGs was involved in the regulation of autophagy, the regulation of JAK-STAT signaling pathway, and the regulation of MAPK cascade. In the protein-protein interaction network, the hub DEGs, such as IL-6, PTGS2, JUN, NQO1, NOS3, LEPR, NAMPT, CDKN2A, CDKN1A, and Snai1, were obtained. After validation using qPCR analysis in the MI mice model and single-cell sequencing, the 10 hub genes can be the potential targets for MI deterioration.

**Conclusion:**

The screened hub genes, IL-6, PTGS2, JUN, NQO1, NOS3, LEPR, NAMPT, CDKN2A, CDKN1A, and Snai1, may be therapeutic targets for patients with MI and may prevent adverse cardiovascular events.

## Introduction

Coronary artery disease (CAD) contributes to considerable mortality and morbidity, leading to over one in every seven deaths all over the world (1). The mortality of patients with atherosclerosis and acute myocardial infarction (MI) has increased by 5.6-fold in the last 3 decades, and obesity has become the major cause in patients with some chronic diseases, for instance, diabetes and CAD (2, 3). More than one-third of the young patients with type 2 diabetic MI die within 10 years, which is associated with higher long-term cardiovascular-related mortality (2–4).

Ferroptosis, a cell death way correlated to intracellular phospholipid peroxidation, is more immunogenic than apoptosis. Several antioxidants can function as ferroptosis inhibitors, which exert anti-inflammatory effects (5, 6). Several metabolic pathways, for example, fatty acid metabolism and mitochondrial respiration, directly impact the cells’ sensitivity toward ferroptosis (7–9). The previous report revealed that the glutathione/GPX4-independent axis impedes ferroptosis and regulates mitochondria quality (9, 10).

Ferritinophagy, a type of ferritin-related autophagy, initiates ferroptosis through the degradation of ferritin (11). Hypoxia impedes ferritinophagy and promotes ferroptosis through NCOA4 expression and c-JUN regulation (12, 13). Ferritinophagy played a critical role in zinc-oxide-nanoparticles-induced ferroptosis of vascular endothelial cells (14).

Obesity plays an important role in T2DM and CAD, especially in MI. Ferroptosis is related to metabolic pathways, such as fatty acid metabolism and mitochondrial respiration. However, insufficient data searches and analytical strategies limited the evidence that supports the functions of ferroptosis-related genes in patients with CAD progression. In this study, GSE116250 was analyzed to explore differentially expressed genes (DEGs) in patients with ischemic cardiomyopathy. A Venn diagram was utilized to obtain the overlapping ferroptosis-and autophagy-related DEGs and further pathway enrichment analyses was carried out. The protein-protein interaction (PPI) network was utilized to obtain the hub genes, which may be the potential ferroptosis- and autophagy-related biomarkers and targets for MI progression. The qPCR analysis in the MI mice model and the single-cell sequencing analysis were used to validate the hub genes’ effects on cardiac functions.

## Materials and Methods

### Animals

Adult experimental C57Bl/6J male mice were purchased from Charles River Laboratories International Inc. (Beijing, China). Mice were maintained in an SPF environment with free access to food and water and a 12/12 light-dark cycle. Protocols were approved by Nankai University and Nankai University Affiliated Third Center Hospital.

The animals were randomly assigned into four groups: (1) Sham (*n* = 4): mice that underwent surgery without ligation; (2) MI (*n* = 6): the MI group, mice that received left artery descending ligation; (3) MI+Alpha-lipoic acid (*n* = 7): mice that received additional Alpha-lipoic acid (2 mM, 0.2ml, ip.); and (4) MI+di ammonium glycyrrhizinate (*n* = 7): mice that received additional di ammonium glycyrrhizinate (1 mM, 0.1 ml, ip.). The two protective drugs were used to explore whether the hub gene expressions can be rescued after MI and with the drug treatment.

### Myocardial Infarction

Myocardial infarction was induced in adult mice (10–11 weeks). Through inhalation of isoflurane (1.5–2%, MSS-3, England), the left coronary artery was ligated and infarction was considered successful following an ST elevation on the electrocardiogram. Sham-operated animals underwent the same procedure without any coronary artery ligation.

### Data Source

Using the keywords “left ventricle” and “Homo sapiens,” GSE116250 was screened out, which was contributed by Sweet ME et al. There are 14 non-failing donors and 13 ischemic cardiomyopathy samples from human left ventricle tissue based on the Illumina HiSeq 2500 (Homo sapiens) (15).

### Differential Expression Analysis

Utilizing the limma package, GSE116250 was explored to investigate DEGs. A log2| (Fold Change) (FC)| > 1 and adjusted *p*-value < 0.05 were regarded as significant.

Total two datasets, including 265 genes from the Ferroptosis Database (FerrDb^1^) and 552 genes from the Autophagy Database (HAMdb^2^), were used and we intersected them with GSE116250 to explore ferroptosis- and autophagy-related DEGs.

### Enrichment Analyses

GO/KEGG pathway analysis (16, 17) were carried out using Metascape^3^ and cluster Profiler Package in R using the Xiantao website.^4^ The Gene Set Enrichment Analysis (GSEA) analysis was performed using WebGestalt.^5^ An adjusted *p-value* < 0.05 was considered significant.

### Protein-Protein Interaction Network Analysis and the Hub Genes

Using STRING (version 11)^6^ and Cytoscape v.3.7.1plug-in (MCODE and MCC), the hub genes were obtained (18, 19). The significant PPIs were identified with a combined score > 0.4. The GO and KEGG pathway analysis were also utilized to understand the functions of hub genes.

### The Hub Genes and Their Interactions

The hub genes and their interactions were analyzed using Network Analyst 3.0.^7^ Specifically, the transcription factor (TF)-screened gene interaction was shown utilizing ENCODE ChIP-seq data (peak intensity signal < 500 and the predicted regulatory potential score < 1). miRNA interactions with the screened hub genes were shown using miRTarBase v8.0. The hub DEG-drug interactions were shown using the DrugBank database (Version 5.0).

### Evaluation of Immune Cells

The xCell can digitally portray the tissue cellular heterogeneity landscape (20). To obtain the abundance of stroma and immune cells in the plaques, the xCell analysis was applied in the GSE116250 dataset. The correlation analysis was used with the Spearman analysis.

### qRT-PCR Analysis

On day 3 and day 7 after MI, quantitative real-time polymerase chain reaction (qRT-PCR) of the left ventricle was performed using the TRIzol method and TB Green^®^ Premix Ex Taq*™* (TaKaRa, RR820). GAPDH was used as a positive control and the 2^–ΔΔCt^ method was used. The primer details were shown in Supplementary Table 1.

### Single-Cell Sequencing Analysis

The single-cell sequencing data about the hub DEGs were used from Single Cell Portal.^8^ The single-cell sequencing data of the human fetal heart, the human heart, and the postnatal mammalian heart from the Single Cell Portal were used to explore the expressions of the hub genes (SCP498, SCP283, and SCP1021) (21–23).

### Statistical Analysis

All of the data were shown as mean ± sem. A one-way ANOVA was used for statistical analysis and a *p* < 0.05 was considered as significant.

## Results

### Identification of Differentially Expressed Genes in GSE114695

**FIGURE 1:**
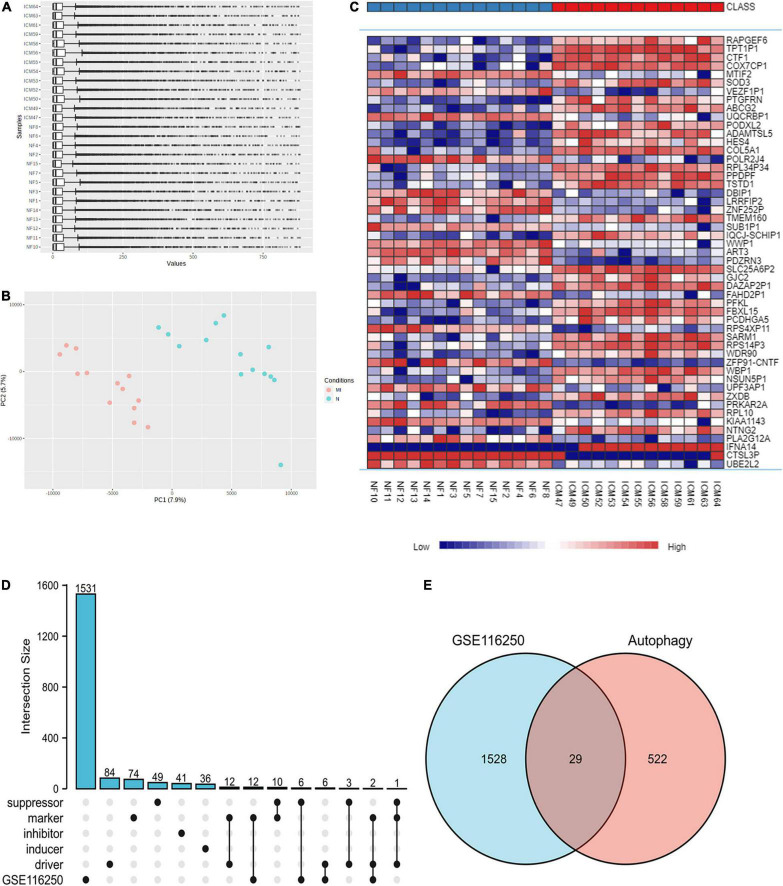
Identification of DEGs in the ischemic myocardiopathy GEO dataset. **(A)** The normalization of the ischemic myocardiopathy dataset was applied through Log2 Transformation. **(B)** The gene cluster in PCA loading score. MI, ischemic myocardiopathy; N, control. **(C)** The heat map of the dataset demonstrated distinguished features between ischemic myocardiopathy and control heart samples. **(D)** An Upset diagram ofGSE116250 DEGs as well as ferroptosis driver, inducer, inhibitor, marker, and suppressor. **(E)** A Venn diagram of GSE116250 DEGs and autophagy-related genes.

Utilizing the limma package, 3237 upregulated and 2012 downregulated DEGs were obtained in GSE116250 after Log^2^ transformation (Figures 1A–C and Supplementary Figure 1). Using the Upset diagram and the Venn diagram, 26 ferroptosis-related and 29 autophagy-related DEGs were obtained (Figures 1D,E, Supplementary Figure 2 and Supplementary Tables 2–4).

### Functional Enrichment Analysis

**FIGURE 2:**
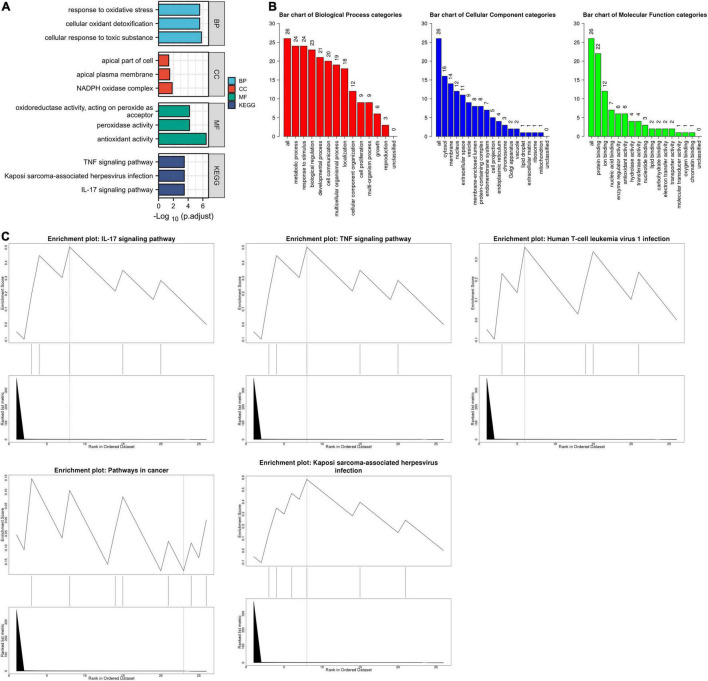
The enrichment pathway analysis of ferroptosis-related DEGs. **(A)** The significant GO and KEGG pathways enriched by ferroptosis-related DEGs using the Xiantao Website. **(B)** The GO enrichment pathway analysis of ferroptosis-related DEGs using WebGestalt. **(C)** The Gene Set Enrichment Analysis of ferroptosis-related DEGs using WebGestalt.

**FIGURE 3:**
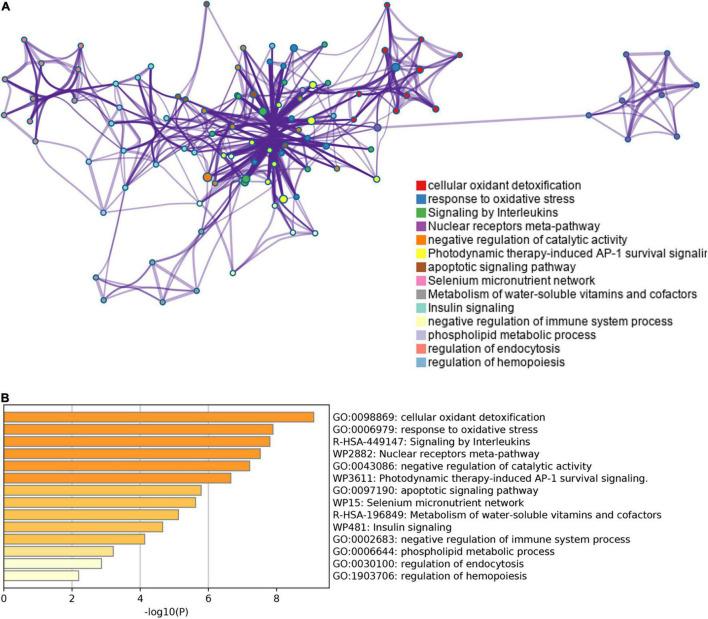
The enrichment pathway analysis of ferroptosis-related DEGs. **(A)** The pathways network of ferroptosis-related DEGs using Metascape. **(B)** The enrichment pathway analysis of ferroptosis-related DEGs using Metascape.

The list of ferroptosis-related DEGs was uploaded into the Xiantao webpage and Metascape for functional analysis, which was involved in the cellular response to toxic substances, the cellular response to toxic cellular oxidant detoxification, the response to oxidative stress, and the response to antioxidant activity. Besides, KEGG enrichment analysis and GSEA both demonstrated that the pathways correlated to ferroptosis-related DEGs mainly included the IL-17 signaling pathway and the TNF signaling pathway (Figures 2, 3, Supplementary Figure 3, and Supplementary Table 5).

**FIGURE 4:**
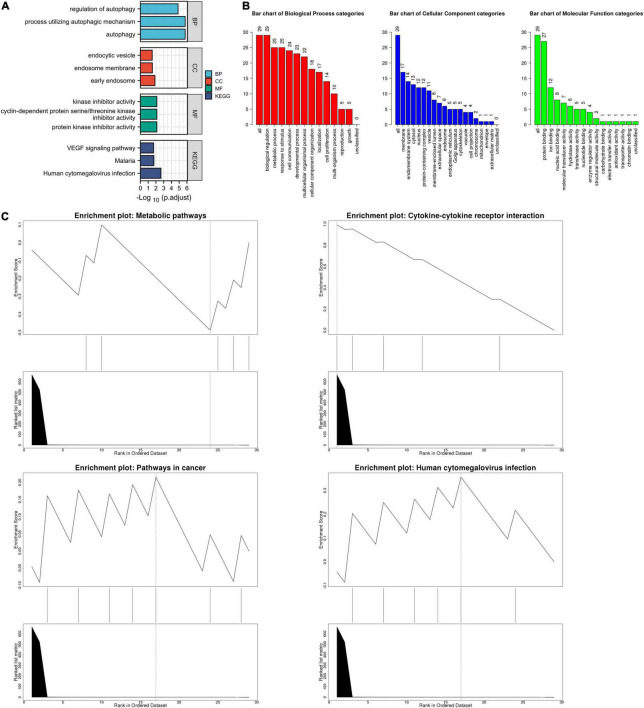
The enrichment pathway analysis of autophagy-related DEGs. **(A)** The significant GO and KEGG pathways enriched by autophagy-related DEGs using the Xiantao Website. **(B)** The GO enrichment pathway analysis of autophagy-related DEGs using WebGestalt. **(C)** The Gene Set Enrichment Analysis of autophagy-related DEGs using WebGestalt.

**FIGURE 5:**
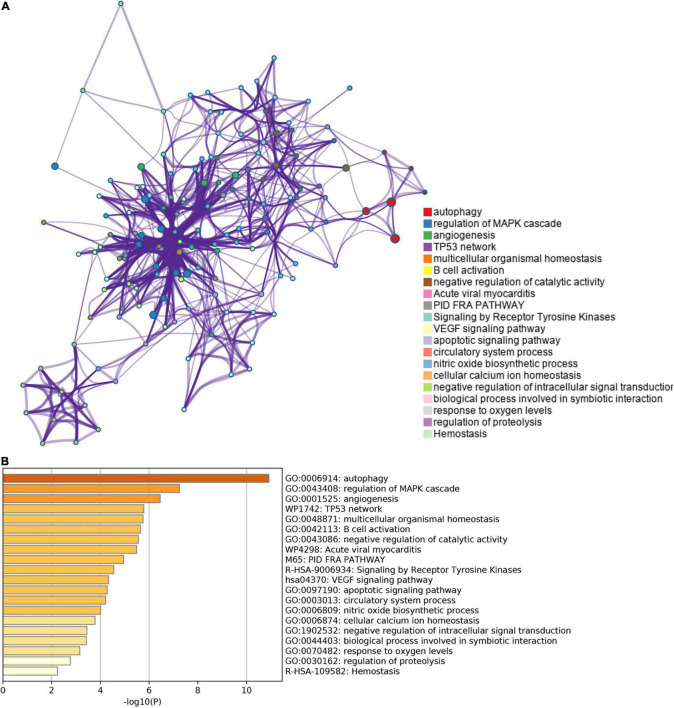
The enrichment pathway analysis of autophagy-related DEGs. **(A)** The pathways network of autophagy-related DEGs using Metascape. **(B)** The enrichment pathway analysis of autophagy-related DEGs using Metascape.

The list of autophagy-related DEGs was also uploaded into the Xiantao webpage and Metascape for functional analyses, which were involved in autophagy, a process utilizing autophagic mechanisms, regulation of autophagy, and regulation of MAPK cascade. Besides, KEGG enrichment analysis demonstrated that the pathways correlated to autophagy-related DEGs mainly included cytokine-cytokine receptor interaction, the JAK-STAT signaling pathway, and cytomegalovirus infection. GSEA demonstrated that the pathways were mainly involved in metabolic pathways and cytokine-cytokine receptor interaction (Figures 4, 5, Supplementary Figure 4, and Supplementary Table 6).

### Protein-Protein Interaction Network Analysis

**FIGURE 6:**
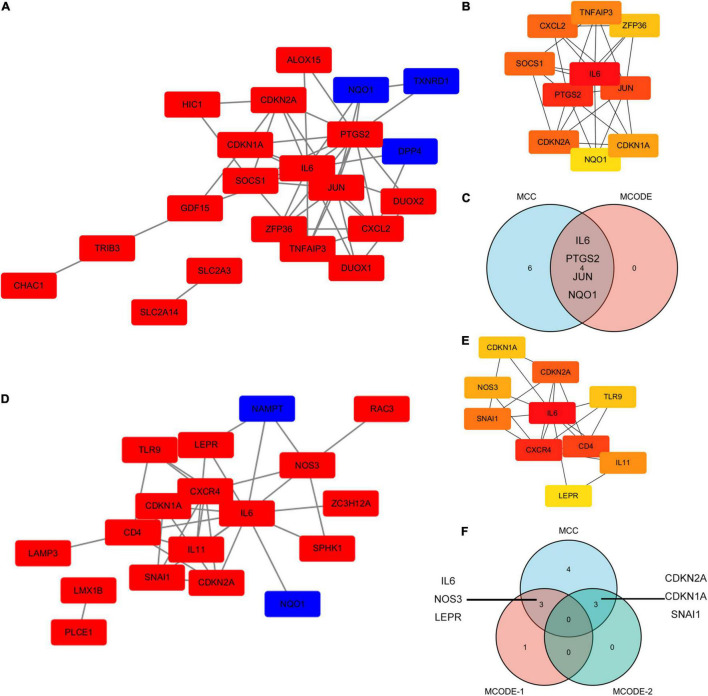
The PPI analysis of ferroptosis-related DEGs and autophagy-related DEGs. **(A)** The PPI analysis of ferroptosis-related DEGs. Red, upregulated genes; blue, downregulated genes. **(B)** The top10 ferroptosis-related DEGs using MCC. **(C)** A Venn diagram of ferroptosis-related DEGs using MCC and MCODE analysis module. **(D)** The PPI analysis of autophagy-related DEGs. Red, upregulated genes; blue, downregulated genes. **(E)** The top 10 autophagy-related DEGs using MCC. **(F)** A Venn diagram of autophagy-related DEGs using MCC and the MCODE analysis module.

The PPI network of ferroptosis- and autophagy-related DEGs was constructed utilizing STRING and Cytoscapev.3.7.1. In the PPI network of ferroptosis-related DEGs, IL-6, PTGS2, JUN, and NQO1 were demonstrated as the hub genes using the Cytoscape plug-in (MCODE and MCC) (Figures 6A–C). In the PPI network of autophagy-related DEGs, there were two MCODEs, such as IL-6, NOS3, LEPR, and NAMPT as well as CDKN2A, CDKN1A, and SNAI1 (Figures 6D–F). In addition, all the ferroptosis- and autophagy-related DEGs together were also analyzed in Supplementary Figure 5.

**FIGURE 7:**
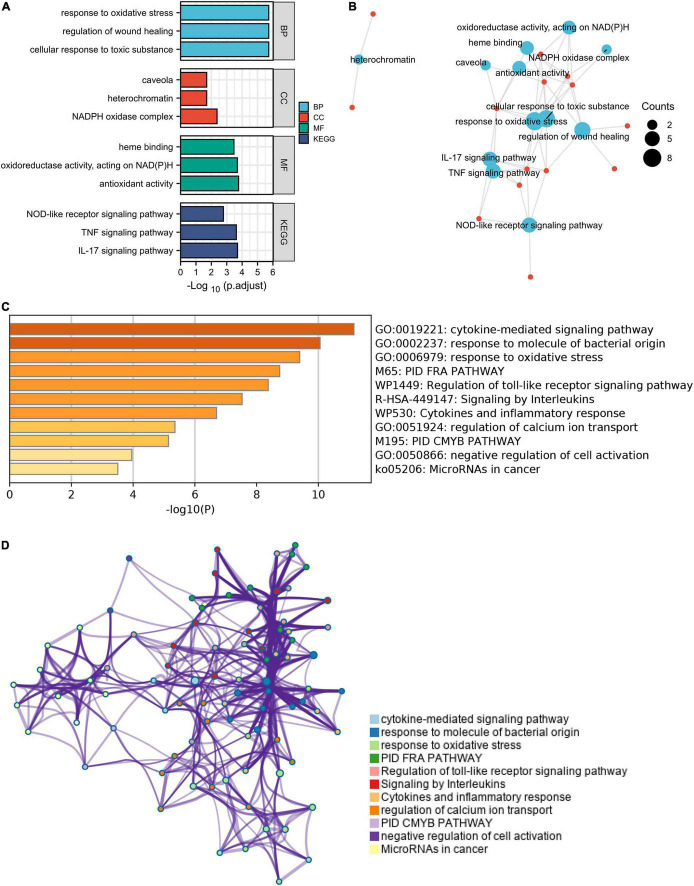
The enrichment pathway analysis of ferroptosis- and autophagy-related hub genes. **(A)** The significant GO and KEGG pathways enriched by ferroptosis- and autophagy-related hub genes using Xiantao Website. **(B)** The pathways network of the hub genes using the Xiantao Website. **(C)** The enrichment pathway analysis of the hub genes using Metascape. **(D)** The pathways network of the hub genes using Metascape.

These ferroptosis- and autophagy-related hub genes were involved in GO such as the regulation of wound healing, responsiveness to oxidative stress, smooth muscle cell proliferation, and oxidoreductase activity, acting on NAD(P)H. Besides, KEGG enrichment analysis demonstrated that the signaling pathways correlated to the hub genes were mainly involved in IL-17 signaling pathway, TNF signaling pathway, and NOD-like receptor signaling pathway (Figure 7 and Supplementary Table 7).

### The Hub Genes and Their Interactions

The hub genes and their interactions were analyzed using Network Analyst 3.0. TF interactions and miRNA interactions with hub genes were shown in Supplementary Figures 6, 7. The hub gene-drug interactions were also analyzed, which demonstrated that the drugs of the targets, for instance, PTGS2, JUN, NOS3, LEPR, and NAMPT, may be used to impede MI recurrent events (Supplementary Figure 8 and Supplementary Table 8).

### Immune Infiltration Analysis

**FIGURE 8:**
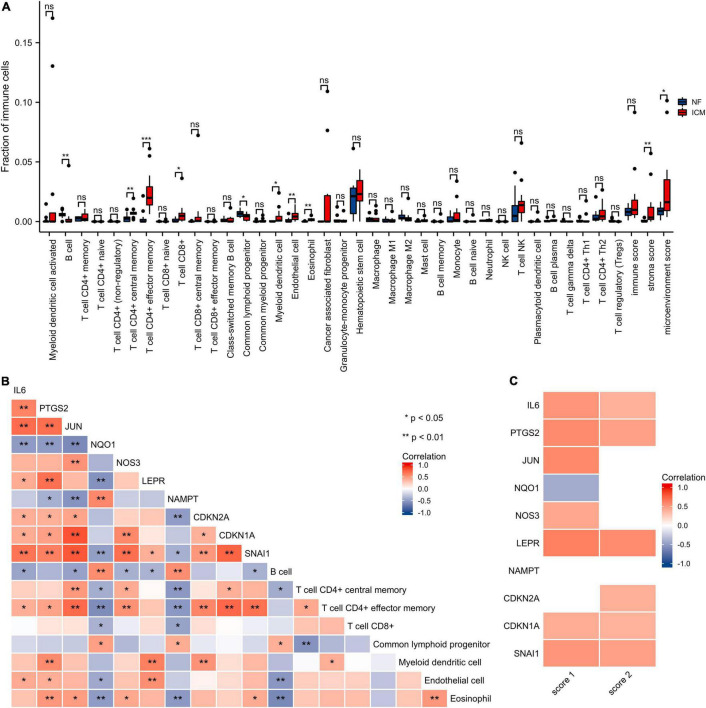
Immune infiltration analysis of the hub genes. **(A)** The score of the immune cells and stroma in GSE116250 is estimated by the xCell. NF, control group; ICM, ischemic myocardiopathy group. **(B)** The correlation heatmap of the hub genes and cell types with differential immune scores. Red, positive correlation; blue, negative correlation. **(C)** The correlation heatmap of the hub genes, stroma score, and microenvironment score. Red, positive correlation; blue, negative correlation; white, *p* > 0.05. Score 1, stroma score; Score 2, microenvironment score.

The xCell analysis was applied to obtain the immune cell compositions in the GSE116250 dataset. Compared with non-failing donors, the left ventricular samples in the ischemic myocardiopathy groups were demonstrated a higher infiltration of T cell CD4+ central memory cells, T cell CD4+ effector memory cells, T cell CD8+ cells, myeloid dendritic cells, endothelial cells, and eosinophil cells and a lower infiltration of B cells and common lymphoid progenitor cells (Figure 8 and Supplementary Table 9).

### Validations of Hub Genes Using qRT-PCR Analysis

**FIGURE 9:**
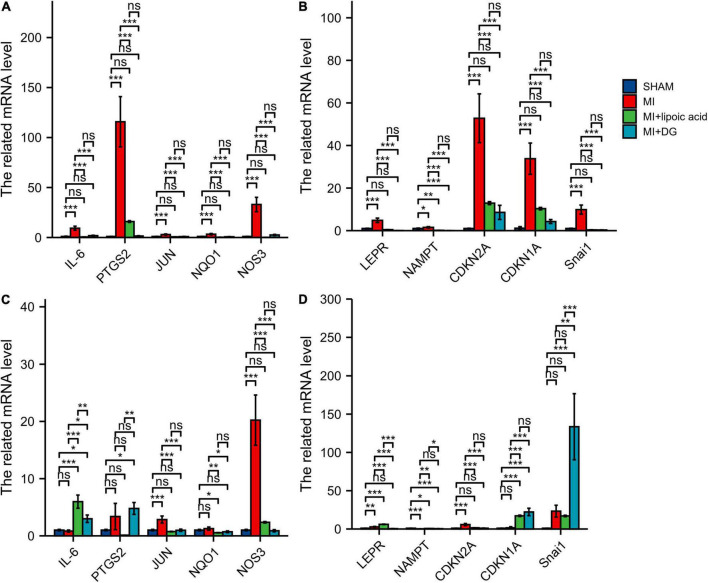
qRT-PCR results show the expression levels of ferroptosis-related and autophagy-related hub genes. **(A)** The related mRNA levels of IL-6, PTGS2, JUN, NQO1, and NOS3 at 3 days after MI. **(B)** The related mRNA levels of LEPR, NAMPT, CDKN2A, CDKN1A, and Snai1 at 3 days after MI. **(C)** The related mRNA levels of IL-6, PTGS2, JUN, NQO1, and NOS3 at 1 week after MI. **(D)** The related mRNA levels of LEPR, NAMPT, CDKN2A, CDKN1A, and Snai1 at 1 week after MI. *p < 0.05; **p < 0.01; ***p < 0.001; ns, not significant.

The hub genes, such as IL-6, PTGS2, JUN, NQO1, NOS3, LEPR, NAMPT, CDKN2A, CDKN1A, and Snai1, were highly expressed at 3 days after MI, and after lipoic acid or diammonium glycyrrhizinate treatment, the hub genes expressions were downregulated (Figures 9A,B). At 1 week after MI, the related mRNA levels of JUN, NOS3, LEPR, NAMPT, CDKN2A, were highly expressed, while there was no significant difference in IL-6, PTGS2, NQO1, CDKN1A, and Snai1 expressions between the MI and SHAM groups. Interestingly, after lipoic acid or diammonium glycyrrhizinate treatment, the IL-6, LEPR, CDKN1A, and Snai1 expressions were highly expressed, which may be other underlying mechanisms (Figures 9C,D).

### Single-Cell Sequencing Analysis

**FIGURE 10:**
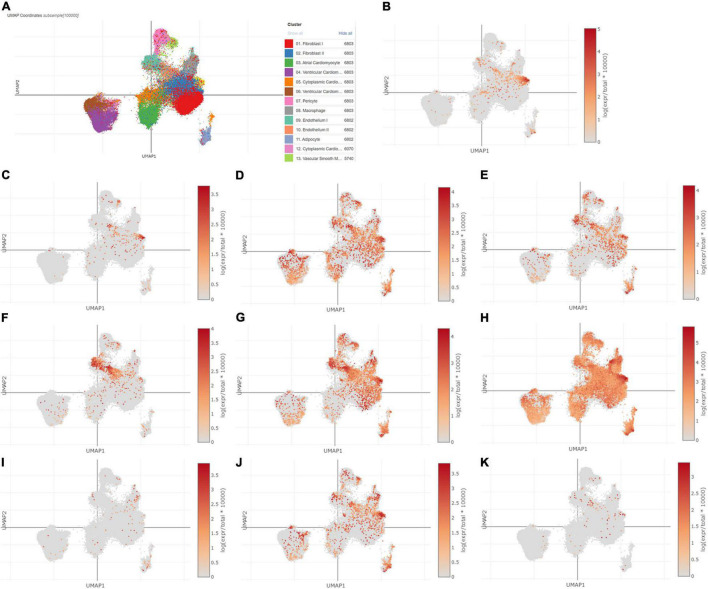
The hub genes were highly expressed in the human heart. **(A)** The overall clustering of cells in the human heart. **(B–K)** The hub genes were validated to be highly expressed in the human heart, such as IL-6 **(B)**, PTGS2 **(C)**, JUN **(D)**, NQO1 **(E)**, NOS3 **(F)**, LEPR **(G)**, NAMPT **(H)**, CDKN2A **(I)**, CDKN1A **(J)**, and Snai1 **(K)**.

**FIGURE 11:**
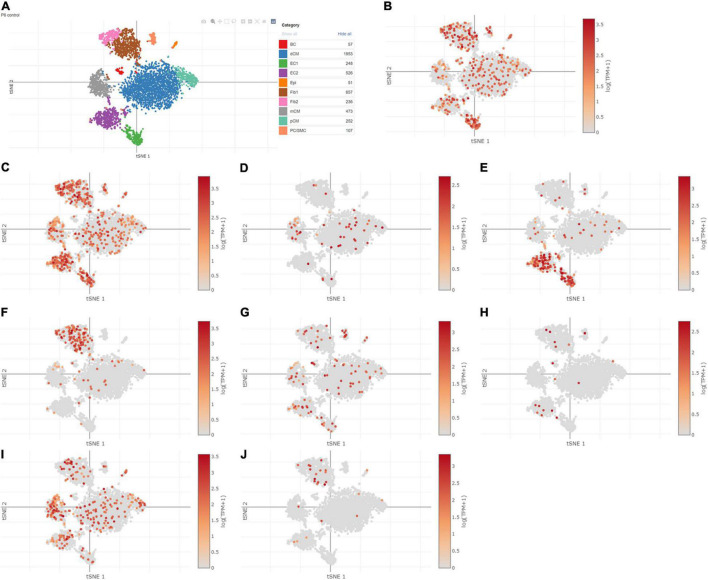
The hub genes were highly expressed in the postnatal mammalian heart. **(A)** The overall clustering of cells in the postnatal mammalian heart. **(B–J)** The hub genes were validated to be highly expressed in the postnatal mammalian heart, such as IL-6 **(B)**, JUN **(C)**, NQO1 **(D)**, NOS3 **(E)**, LEPR **(F)**, NAMPT **(G)**, CDKN2A **(H)**, CDKN1A **(I)**, and Snai1 **(J)**, while there was no PTGS2 expression in the postnatal mammalian heart.

**FIGURE 12:**
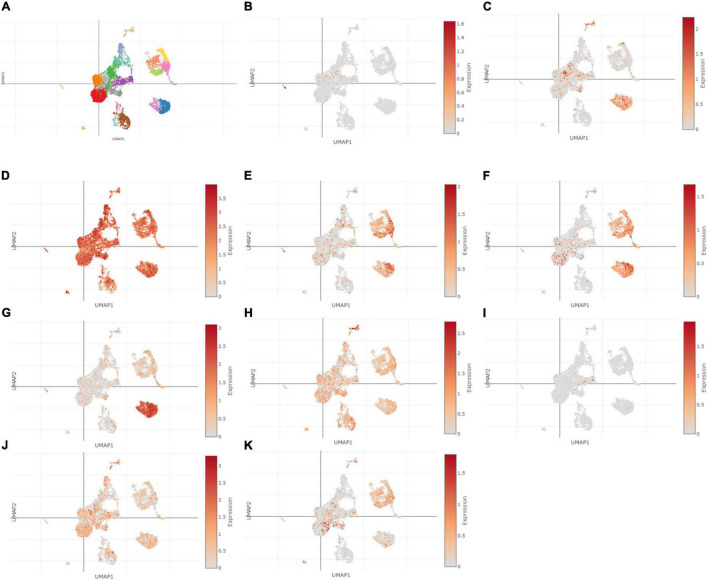
The hub genes were highly expressed in the human fetal heart. **(A)** The overall clustering of cells in the human fetal heart. **(B–K)** The hub genes were validated to be highly expressed in the fetal human heart, such as IL-6 **(B)**, PTGS2 **(C)**, JUN **(D)**, NQO1 **(E)**, NOS3 **(F)**, LEPR **(G)**, NAMPT **(H)**, CDKN2A **(I)**, CDKN1A **(J)**, and Snai1 **(K)**.

Using Single Cell Portal, single-cell sequencing of the hub genes expression in the heart was also investigated, suggesting that the hub genes were highly expressed in the human fetal heart, the human heart, and the postnatal mammalian heart (Figures 10–12). Interestingly, there were no PTGS2 expressions in the postnatal mammalian heart.

## Discussion

The etiology of MI progression is relatively clear. MI promotes atherosclerosis progression by the release of progenitor cells and hematopoietic stem cells from the bone marrow niche (4, 24). An endothelial vasomotor function can be consistently impaired when patients develop ST-segment elevation myocardial infarction (STEMI), which is highly correlated to atherosclerosis development and plaque progression (25). However, the prognostic and diagnostic biomarkers and treatment targets of MI progression are still well-unknown.

In this study, we obtained ferroptosis- and autophagy-related DEGs in GSE116250, and further analysis and validations were used as well. Acute or chronic cellular stress results in considerable non-apoptotic cell death (ferroptosis) (7, 8). The ferroptosis-related DEGs were involved in regards to the cellular response to toxic substances, the cellular response to cellular oxidant detoxification, the response to oxidative stress, and the response to antioxidant activity (26–28). Besides, KEGG enrichment analysis demonstrated that the pathways correlated to ferroptosis-related DEGs also included the IL-17 signaling pathway, which was rarely reported previously. The list of autophagy-related DEGs was involved in the regulation of autophagy, MAPK cascade, the JAK-STAT signaling pathway, and cytomegalovirus infection (29–31). Although ferroptosis-related DEGs still found ferroptosis-related functions and autophagy-related DEGs still found autophagy-related functions, we used a variety of enrichment methods to investigate the different related functions, which were caused by the way of the enrichment methods. The different enriched pathways among the enrichment methods may show the underlying mechanisms and the clue about the crosstalk between ferroptosis and autophagy pathways.

After validation using GEO datasets and MI mice model, the 10 hub genes, such as IL-6, PTGS2, JUN, NQO1, NOS3, LEPR, NAMPT, CDKN2A, CDKN1A, and Snai1, can be the diagnostic ferroptosis- and autophagy-related targets and biomarkers in MI deterioration and can be targets to prevent adverse cardiac events. IL-6, as a protective cytokine, promoted cellular dedifferentiation and led to pericardial ADSC-induced cardiac repair (32). Higher IL-6 levels were independently correlated to the risk of major adverse cardiovascular events (MACE), and elevated IL-6 level was associated with an increased risk of MACE at normal kidney function, mild chronic kidney disease (CKD), and moderate to severe CKD (33). A PTGS2 variant rs20417 (COX-2 encoded) polymorphism can reduce the risk of MACE (34). MicroRNA-26b can bind to PTGS2 and alleviate myocardial injury through the MAPK pathway (35). Nrf2, NQO1, and HO-1, as antioxidative molecules, were always used as biomarkers to detect the drug effects on MI and Parkinson’s disease (36, 37), for instance, cytoplasm HO-1, cytoplasm NQO1, and nucleus Nrf2 expressions increased both *in vivo* and *in vitro* using azafrin after MI. Ischemic postconditioning increased the expression of Nrf2, NQO1, and HO-1 and impeded MI-induced oxidative stress (38). The NOS3 c.894G > T and 27-bp VNTR polymorphisms can be used for CAD screening (39). In the Women’s Genome Health Study, metabolic-syndrome related genes, such as LEPR, HNF1A, IL6R, and GCKR, were correlated to CRP expression and inflammation (40). Type 2 DM mice (C57BLKS/J Lepr (db)/Lepr (db) mice and BKS.Cg-m+/+Lepr(db)/J mice) were used to investigate LEPR effects on cardiomyocyte function and ventricular arrhythmias after MI and after developing diabetes (41, 42). NAMPT and NAMPT-controlled NAD metabolism can regulate vascular repair, which may show protective effects on cardiac regeneration after MI (43). CDKN2A/B (rs10757274) polymorphism was correlated to MI risk and T2DM aggravated MI through the interaction with the polymorphism in Chinese populations and in Middle East populations (44, 45). In addition, megakaryopoiesis and platelet activity increased in hypercholesterolemic, B6-Ldlr-/-, Cdkn2a-Deficient Mice (46). Copper induces cell death by targeting lipoylated TCA cycle proteins through downstream PDH complex, such as CDKN2A expression, and lipoic acid pathway (47). In this study, CDKN2A expression increased after MI and was rescued by the treatment of lipoic acid, which may show some clues to the interaction among ferroptosis, copper-induced cell death, and autophagy. CDKN1a/p21, as an ischemia-reperfusion injury modulator, was associated with the risk of atherosclerosis and MI progression (48, 49). MI-related exosomes delivered miR-208b, which regulated the growth of HUVECs through regulating CDKN1A expression (50). miR-30a/e controls cardiac epithelial to mesenchymal transition (EMT) by targeting the expression of SNAI1 and NOTCH1, and Losartan inhibits EMT in mitral valve endothelial cells by the targets (51, 52). In addition, ferritinophagy initiates ferroptosis through the degradation of ferritin, which controls the crosstalk between ferroptosis and the autophagy pathway (11). Hypoxia impedes ferritinophagy and promotes ferroptosis through NCOA4 expression and c-JUN regulation (12, 13). Ferritinophagy played a critical role in zinc-oxide-nanoparticles-induced ferroptosis of vascular endothelial cells by regulating some EMT- related targets, such as Snai1 (14).

There are some limitations. First, there may be false negatives because of the enrichment methods. The other neighbor genes still need to be investigated. Second, the sample sizes of included datasets were not too large. Further research is still needed to explore the functional effects of the screened hub genes to help improve the prognosis and prevent MI progression and adverse cardiovascular events.

## Conclusion

Based on our current study, our research provided bioinformatics analysis of ferroptosis- and autophagy-related DEGs. The screened hub genes, IL-6, PTGS2, JUN, NQO1, NOS3, LEPR, NAMPT, CDKN2A, CDKN1A, and Snai1, may be therapeutic targets to prevent MI progression and adverse cardiovascular events.

## Data Availability Statement

The datasets presented in this study can be found in online repositories. The names of the repository/repositories and accession number(s) can be found in the article/Supplementary Material.

## Ethics Statement

The animal study was reviewed and approved by Nankai University and Nankai University Affiliated Third Center Hospital.

## Author Contributions

YZ conceived the idea. YZ, WG, and XC downloaded the data and carried out further analysis. YZ, QZ, YL, and ZQ visualized the results. YZ wrote the manuscript. YZ and TL supervised the manuscript. All authors contributed to the article and approved the submitted version.

## Conflict of Interest

The authors declare that the research was conducted in the absence of any commercial or financial relationships that could be construed as a potential conflict of interest.

## Publisher’s Note

All claims expressed in this article are solely those of the authors and do not necessarily represent those of their affiliated organizations, or those of the publisher, the editors and the reviewers. Any product that may be evaluated in this article, or claim that may be made by its manufacturer, is not guaranteed or endorsed by the publisher.

## Footnotes

1
zhounan.org

2
scbdd.com

3
https://metascape.org/gp/index.html#/main/step1

4
www.xiantao.love

5
http://www.webgestalt.org/

6
https://cn.string-db.org/

7
https://www.networkanalyst.ca/

8
https://singlecell.broadinstitute.org/

## Supplementary Material

Supplementary Figure 1 The PPI network of the DEGs in GSE116250.

Supplementary Figure 2 A Venn diagram of DEGs and ferroptosis genes.

Supplementary Figure 3 The network of the enriched pathways of ferroptosis-related genes.

Supplementary Figure 4 The network of the enriched pathways of autophagy-related genes.

Supplementary Figure 5 The PPI analysis of ferroptosis-related and autophagy-related DEGs together. **(A)** The PPI analysis of ferroptosis-related and autophagy-related DEGs together. Red, upregulated genes; blue, downregulated genes. **(B)** The top 20 of the DEGs mentioned above using MCC. **(C)** A Venn diagram of the DEGs mentioned above using MCC and MCODE analysis module.

Supplementary Figure 6 The ferroptosis-related hub genes and their interactions. **(A)** miRNA coregulatory interactions with the screened hub genes. **(B)** TF coregulatory interactions with the screened hub genes.

Supplementary Figure 7 The autophagy-related hub genes and their interactions. **(A)** miRNA interactions with the MCODE-1 hubgenes. **(B)** miRNA interactions with the MCODE-2 hubgenes. **(C)** TF interactions with the MCODE-1 hub genes. **(D)** TF interactions with the MCODE-2 hub genes.

Supplementary Figure 8 The hub genes and their druginteractions. **(A)** The ferroptosis-related hub genes and their druginteractions. **(B)** The autophagy-related hub genes and their drug interactions.
