# Supplementary material for: Ferroptosis and Autophagy-Related Genes in the Pathogenesis of Ischemic Cardiomyopathy
Source: Front Cardiovasc Med. 2022 Jun 30;9:906753. doi: 10.3389/fcvm.2022.906753 (PMC9279674; doi:10.3389/fcvm.2022.906753)
Supplement: Supplementary file 1 [file Data_Sheet_1.docx]

Supplementary Table 1. Specific primers used for quantitative real-time PCR.

| Genes | Forward (5’-3’) | Reverse (5’-3’) |
| --- | --- | --- |
| IL6 | TAGTCCTTCCTACCCCAATTTCC | TTGGTCCTTAGCCACTCCTTC |
| PTGS2 | TTCAACACACTCTATCACTGGC | AGAAGCGTTTGCGGTACTCAT |
| JUN | CCTTCTACGACGATGCCCTC | GGTTCAAGGTCATGCTCTGTTT |
| NQO1 | AGGATGGGAGGTACTCGAATC | AGGCGTCCTTCCTTATATGCTA |
| NOS3 | GGCTGGGTTTAGGGCTGTG | CTGAGGGTGTCGTAGGTGATG |
| LEPR | GTCTTCGGGGATGTGAATGTC | ACCTAAGGGTGGATCGGGTTT |
| NAMPT | GCAGAAGCCGAGTTCAACATC | TTTTCACGGCATTCAAAGTAGGA |
| CDKN2A | CGCAGGTTCTTGGTCACTGT | TGTTCACGAAAGCCAGAGCG |
| CDKN1A | CCTGGTGATGTCCGACCTG | CCATGAGCGCATCGCAATC |
| SNAI1 | CACACGCTGCCTTGTGTCT | GGTCAGCAAAAGCACGGTT |
| B-actin | GGCTGTATTCCCCTCCATCG | CCAGTTGGTAACAATGCCATGT |

Supplementary Table 2. Ferroptosis-related differentially expressed genes of ischemic cardiomyopathy.

| Gene symbol | Adj. P value | logFC | Gene title | EntrezID |
| --- | --- | --- | --- | --- |
| Upregulated genes | | | | |
| HBA1 | 0.00033019 | 2.8843 | hemoglobin subunit alpha 1 | 3039 |
| JUN | 0.00041491 | 1.1944 | "Jun proto-oncogene, AP-1 transcription factor subunit" | 3725 |
| PROM2 | 0.0016288 | 1.0471 | prominin 2 | 150696 |
| ENPP2 | 0.0018358 | 1.1655 | ectonucleotide pyrophosphatase/phosphodiesterase 2 | 5168 |
| CXCL2 | 0.0018826 | 2.1743 | C-X-C motif chemokine ligand 2 | 2920 |
| HIC1 | 0.0022144 | 1.034 | HIC ZBTB transcriptional repressor 1 | 3090 |
| TRIB3 | 0.0023324 | 1.1286 | tribbles pseudokinase 3 | 57761 |
| DUOX1 | 0.0025335 | 1.348 | dual oxidase 1 | 53905 |
| GDF15 | 0.0035746 | 2.0338 | growth differentiation factor 15 | 9518 |
| DUOX2 | 0.0036234 | 1.1425 | dual oxidase 2 | 50506 |
| ZFP36 | 0.0042057 | 1.8109 | ZFP36 ring finger protein | 7538 |
| SLC2A14 | 0.0061225 | 1.6067 | solute carrier family 2 member 14 | 144195 |
| SOCS1 | 0.0066991 | 1.4976 | suppressor of cytokine signaling 1 | 8651 |
| PTGS2 | 0.0075026 | 1.7452 | prostaglandin-endoperoxide synthase 2 | 5743 |
| IL6 | 0.0077121 | 2.6019 | interleukin 6 | 3569 |
| SLC2A3 | 0.0096906 | 1.4618 | solute carrier family 2 member 3 | 6515 |
| CDKN2A | 0.0097604 | 1.2882 | cyclin dependent kinase inhibitor 2A | 1029 |
| CHAC1 | 0.011945 | 1.7501 | ChaC glutathione specific gamma-glutamylcyclotransferase 1 | 79094 |
| TNFAIP3 | 0.019852 | 1.0957 | TNF alpha induced protein 3 | 7128 |
| CDKN1A | 0.030085 | 1.0856 | cyclin dependent kinase inhibitor 1A | 1026 |
| RGS4 | 0.033052 | 1.6856 | regulator of G protein signaling 4 | 5999 |
| ALOX15 | 0.042146 | 375.81 | arachidonate 15-lipoxygenase | 246 |
| NNMT | 0.048418 | 1.1207 | nicotinamide N-methyltransferase | 4837 |
| Downregulated genes | | | | |
| TXNRD1 | 0.0055479 | -1.0216 | thioredoxin reductase 1 | 7296 |
| DPP4 | 0.0011475 | -1.1856 | dipeptidyl peptidase 4 | 1803 |
| NQO1 | 0.000023239 | -1.3846 | NAD(P)H quinone dehydrogenase 1 | 1728 |

FC, fold change.

Supplementary Table 3. The ferroptosis-related differentially expressed genes were divided into ferroptosis driver, marker and suppressor.

| driver | inducer | inhibitor | marker | suppressor |
| --- | --- | --- | --- | --- |
| DPP4  DUOX2  ALOX15  TNFAIP3  CHAC1  DUOX1  SOCS1  CDKN2A | / | / | GDF15  TRIB3  SLC2A3  RGS4  ALOX15  PTGS2  NNMT  IL6  CHAC1  SLC2A14  HIC1  CXCL2  TXNRD1  HBA1 | PROM2  ZFP36  ENPP2  NQO1  CDKN1A  JUN |

Supplementary Table 4. Autophagy-related differentially expressed genes of ischemic cardiomyopathy.

| Gene symbol | Adj. P value | logFC | Gene title | EntrezID |
| --- | --- | --- | --- | --- |
| Upregulated genes | | | | |
| MAP1S | 0.000006607 | 1.1336 | microtubule associated protein 1S | 55201 |
| PLCE1 | 0.000007735 | 1.3543 | phospholipase C epsilon 1 | 51196 |
| RASIP1 | 8.8384E-06 | 1.1196 | Ras interacting protein 1 | 54922 |
| CLEC11A | 0.000092819 | 1.8239 | C-type lectin domain containing 11A | 6320 |
| CXCR4 | 0.00042693 | 1.5805 | C-X-C motif chemokine receptor 4 | 7852 |
| TLR9 | 0.00066661 | 1.6076 | toll like receptor 9 | 54106 |
| TCIRG1 | 0.0007719 | 1.0441 | "T cell immune regulator 1, ATPase H+ transporting V0 subunit a3" | 10312 |
| SNAI1 | 0.00087436 | 2.2756 | snail family transcriptional repressor 1 | 6615 |
| RAC3 | 0.00099363 | 1.1613 | Rac family small GTPase 3 | 5881 |
| LMX1B | 0.0010846 | 1.2522 | LIM homeobox transcription factor 1 beta | 4010 |
| CD4 | 0.0016932 | 1.253 | CD4 molecule | 920 |
| IL11 | 0.0021852 | 665.34 | interleukin 11 | 3589 |
| TRIB3 | 0.0023324 | 1.1286 | tribbles pseudokinase 3 | 57761 |
| BOK | 0.0028032 | 1.1769 | BCL2 family apoptosis regulator BOK | 666 |
| LEPR | 0.0050901 | 1.1278 | leptin receptor | 3953 |
| THBS2 | 0.0061343 | 1.1639 | thrombospondin 2 | 7058 |
| IL6 | 0.0077121 | 2.6019 | interleukin 6 | 3569 |
| CDKN2A | 0.0097604 | 1.2882 | cyclin dependent kinase inhibitor 2A | 1029 |
| TBC1D10C | 0.013573 | 1.3995 | TBC1 domain family member 10C | 374403 |
| SPHK1 | 0.014514 | 1.4171 | sphingosine kinase 1 | 8877 |
| LAMP3 | 0.016187 | 520.96 | lysosomal associated membrane protein 3 | 27074 |
| NOS3 | 0.018845 | 1.0123 | nitric oxide synthase 3 | 4846 |
| ZC3H12A | 0.024911 | 1.1315 | zinc finger CCCH-type containing 12A | 80149 |
| CDKN1A | 0.030085 | 1.0856 | cyclin dependent kinase inhibitor 1A | 1026 |
| PTGER2 | 0.032457 | 1.2186 | prostaglandin E receptor 2 | 5732 |
| KIF25 | 0.03276 | 1.1541 | kinesin family member 25 | 3834 |
| DACT1 | 0.035091 | 1.022 | dishevelled binding antagonist of beta catenin 1 | 51339 |
| Downregulated genes | | | | |
| NQO1 | 0.000023239 | -1.3846 | NAD(P)H quinone dehydrogenase 1 | 1728 |
| NAMPT | 0.000029391 | -1.6059 | nicotinamide phosphoribosyltransferase | 10135 |

FC, fold change.

Supplementary Table 5. The significant GO and KEGG pathways enriched by ferroptosis-related DEGs.

| ONTOLOGY | ID | Description | GeneRatio | BgRatio | pvalue | p.adjust | qvalue |
| --- | --- | --- | --- | --- | --- | --- | --- |
| BP | GO:0097237 | cellular response to toxic substance | 8/26 | 247/18670 | 1.07e-09 | 1.55e-06 | 9.27e-07 |
| BP | GO:0098869 | cellular oxidant detoxification | 6/26 | 102/18670 | 4.83e-09 | 2.71e-06 | 1.63e-06 |
| BP | GO:0006979 | response to oxidative stress | 9/26 | 451/18670 | 5.61e-09 | 2.71e-06 | 1.63e-06 |
| BP | GO:1990748 | cellular detoxification | 6/26 | 112/18670 | 8.50e-09 | 3.08e-06 | 1.85e-06 |
| BP | GO:0098754 | detoxification | 6/26 | 131/18670 | 2.18e-08 | 6.33e-06 | 3.79e-06 |
| CC | GO:0043020 | NADPH oxidase complex | 2/26 | 15/19717 | 1.74e-04 | 0.015 | 0.011 |
| CC | GO:0016324 | apical plasma membrane | 4/26 | 318/19717 | 7.50e-04 | 0.031 | 0.023 |
| CC | GO:0045177 | apical part of cell | 4/26 | 384/19717 | 0.002 | 0.042 | 0.031 |
| MF | GO:0016209 | antioxidant activity | 6/26 | 86/17697 | 2.35e-09 | 3.59e-07 | 2.13e-07 |
| MF | GO:0004601 | peroxidase activity | 4/26 | 52/17697 | 9.44e-07 | 6.51e-05 | 3.85e-05 |
| MF | GO:0016684 | oxidoreductase activity, acting on peroxide as acceptor | 4/26 | 56/17697 | 1.28e-06 | 6.51e-05 | 3.85e-05 |
| MF | GO:0004860 | protein kinase inhibitor activity | 4/26 | 63/17697 | 2.06e-06 | 7.86e-05 | 4.65e-05 |
| MF | GO:0019210 | kinase inhibitor activity | 4/26 | 67/17697 | 2.63e-06 | 8.06e-05 | 4.77e-05 |
| KEGG | hsa04657 | IL-17 signaling pathway | 5/21 | 94/8076 | 3.37e-06 | 3.33e-04 | 1.91e-04 |
| KEGG | hsa05167 | Kaposi sarcoma-associated herpesvirus infection | 6/21 | 193/8076 | 6.93e-06 | 3.33e-04 | 1.91e-04 |
| KEGG | hsa04668 | TNF signaling pathway | 5/21 | 112/8076 | 7.99e-06 | 3.33e-04 | 1.91e-04 |
| KEGG | hsa05166 | Human T-cell leukemia virus 1 infection | 5/21 | 219/8076 | 2.00e-04 | 0.006 | 0.004 |
| KEGG | hsa05225 | Hepatocellular carcinoma | 4/21 | 168/8076 | 8.20e-04 | 0.021 | 0.012 |

DEGs, Differential Expressed Genes; BP, Biological Process; CC, cellular component; MF, Molecular Function; KEGG, Kyoto Encyclopedia of Genes and Genomes.

Supplementary Table 6. The significant GO and KEGG pathways enriched by autophagy-related DEGs.

| ONTOLOGY | ID | Description | GeneRatio | BgRatio | pvalue | p.adjust | qvalue |
| --- | --- | --- | --- | --- | --- | --- | --- |
| BP | GO:0006914 | autophagy | 10/29 | 496/18670 | 2.04e-09 | 1.70e-06 | 1.01e-06 |
| BP | GO:0061919 | process utilizing autophagic mechanism | 10/29 | 496/18670 | 2.04e-09 | 1.70e-06 | 1.01e-06 |
| BP | GO:0010506 | regulation of autophagy | 8/29 | 327/18670 | 2.53e-08 | 1.41e-05 | 8.35e-06 |
| BP | GO:0030098 | lymphocyte differentiation | 7/29 | 353/18670 | 8.88e-07 | 3.71e-04 | 2.19e-04 |
| BP | GO:1900119 | positive regulation of execution phase of apoptosis | 3/29 | 15/18670 | 1.51e-06 | 5.07e-04 | 2.99e-04 |
| CC | GO:0005769 | early endosome | 5/29 | 350/19717 | 1.43e-04 | 0.013 | 0.009 |
| CC | GO:0010008 | endosome membrane | 5/29 | 479/19717 | 6.08e-04 | 0.029 | 0.020 |
| CC | GO:0030139 | endocytic vesicle | 4/29 | 303/19717 | 9.59e-04 | 0.030 | 0.021 |
| CC | GO:0101003 | ficolin-1-rich granule membrane | 2/29 | 61/19717 | 0.004 | 0.085 | 0.058 |
| CC | GO:0000792 | heterochromatin | 2/29 | 78/19717 | 0.006 | 0.097 | 0.066 |
| MF | GO:0004860 | protein kinase inhibitor activity | 3/28 | 63/17697 | 1.32e-04 | 0.008 | 0.005 |
| MF | GO:0004861 | cyclin-dependent protein serine/threonine kinase inhibitor activity | 2/28 | 12/17697 | 1.58e-04 | 0.008 | 0.005 |
| MF | GO:0019210 | kinase inhibitor activity | 3/28 | 67/17697 | 1.59e-04 | 0.008 | 0.005 |
| MF | GO:0016653 | oxidoreductase activity, acting on NAD(P)H, heme protein as acceptor | 2/28 | 13/17697 | 1.86e-04 | 0.008 | 0.005 |
| MF | GO:0004896 | cytokine receptor activity | 3/28 | 96/17697 | 4.59e-04 | 0.015 | 0.010 |
| KEGG | hsa05163 | Human cytomegalovirus infection | 6/21 | 225/8076 | 1.67e-05 | 0.002 | 0.002 |
| KEGG | hsa05144 | Malaria | 3/21 | 50/8076 | 2.75e-04 | 0.018 | 0.013 |
| KEGG | hsa04370 | VEGF signaling pathway | 3/21 | 59/8076 | 4.49e-04 | 0.020 | 0.014 |
| KEGG | hsa04630 | JAK-STAT signaling pathway | 4/21 | 162/8076 | 7.15e-04 | 0.020 | 0.014 |
| KEGG | hsa04060 | Cytokine-cytokine receptor interaction | 5/21 | 295/8076 | 7.90e-04 | 0.020 | 0.014 |

DEGs, Differential Expressed Genes; BP, Biological Process; CC, cellular component; MF, Molecular Function; KEGG, Kyoto Encyclopedia of Genes and Genomes.

Supplementary Table 7. The significant GO and KEGG pathways enriched by ferroptosis- and autophagy-related DEGs.

| ONTOLOGY | ID | Description | GeneRatio | BgRatio | pvalue | p.adjust | qvalue |
| --- | --- | --- | --- | --- | --- | --- | --- |
| BP | GO:0097237 | cellular response to toxic substance | 7/18 | 247/18670 | 1.83e-09 | 1.87e-06 | 7.74e-07 |
| BP | GO:0061041 | regulation of wound healing | 6/18 | 148/18670 | 3.85e-09 | 1.87e-06 | 7.74e-07 |
| BP | GO:0006979 | response to oxidative stress | 8/18 | 451/18670 | 3.86e-09 | 1.87e-06 | 7.74e-07 |
| BP | GO:0048660 | regulation of smooth muscle cell proliferation | 6/18 | 169/18670 | 8.54e-09 | 2.66e-06 | 1.10e-06 |
| BP | GO:0048659 | smooth muscle cell proliferation | 6/18 | 171/18670 | 9.16e-09 | 2.66e-06 | 1.10e-06 |
| CC | GO:0043020 | NADPH oxidase complex | 2/18 | 15/19717 | 8.21e-05 | 0.004 | 0.002 |
| CC | GO:0000792 | heterochromatin | 2/18 | 78/19717 | 0.002 | 0.020 | 0.011 |
| CC | GO:0005901 | caveola | 2/18 | 80/19717 | 0.002 | 0.020 | 0.011 |
| CC | GO:0005788 | endoplasmic reticulum lumen | 3/18 | 309/19717 | 0.003 | 0.020 | 0.011 |
| CC | GO:0045121 | membrane raft | 3/18 | 315/19717 | 0.003 | 0.020 | 0.011 |
| MF | GO:0016209 | antioxidant activity | 4/18 | 86/17697 | 1.51e-06 | 1.71e-04 | 7.79e-05 |
| MF | GO:0016651 | oxidoreductase activity, acting on NAD(P)H | 4/18 | 107/17697 | 3.62e-06 | 2.05e-04 | 9.34e-05 |
| MF | GO:0020037 | heme binding | 4/18 | 135/17697 | 9.12e-06 | 3.42e-04 | 1.56e-04 |
| MF | GO:0046906 | tetrapyrrole binding | 4/18 | 145/17697 | 1.21e-05 | 3.42e-04 | 1.56e-04 |
| MF | GO:0004601 | peroxidase activity | 3/18 | 52/17697 | 1.89e-05 | 4.28e-04 | 1.95e-04 |
| KEGG | hsa04657 | IL-17 signaling pathway | 5/18 | 94/8076 | 1.46e-06 | 2.00e-04 | 1.04e-04 |
| KEGG | hsa04668 | TNF signaling pathway | 5/18 | 112/8076 | 3.48e-06 | 2.38e-04 | 1.25e-04 |
| KEGG | hsa04621 | NOD-like receptor signaling pathway | 5/18 | 181/8076 | 3.62e-05 | 0.002 | 8.63e-04 |
| KEGG | hsa05167 | Kaposi sarcoma-associated herpesvirus infection | 5/18 | 193/8076 | 4.92e-05 | 0.002 | 8.81e-04 |
| KEGG | hsa05166 | Human T-cell leukemia virus 1 infection | 5/18 | 219/8076 | 8.99e-05 | 0.002 | 0.001 |

DEGs, Differential Expressed Genes; BP, Biological Process; CC, cellular component; MF, Molecular Function; KEGG, Kyoto Encyclopedia of Genes and Genomes.

Supplementary Table 8. The detailed information of hub genes.

| **Hub genes** | **Full name** | **Functions** |
| --- | --- | --- |
| IL6 | Interleukin-6 | Cytokine with a wide variety of biological functions. It is a potent inducer of the acute phase response. Plays an essential role in the final differentiation of B-cells into Ig- secreting cells Involved in lymphocyte and monocyte differentiation. Acts on B-cells, T-cells, hepatocytes, hematopoietic progenitor cells and cells of the CNS. Required for the generation of T(H)17 cells. |
| PTGS2 | Prostaglandin G/H synthase 2 | Converts arachidonate to prostaglandin H2 (PGH2), a committed step in prostanoid synthesis. Constitutively expressed in some tissues in physiological conditions, such as the endothelium, kidney and brain, and in pathological conditions |
| JUN | Transcription factor AP-1 | Transcription factor that recognizes and binds to the enhancer heptamer motif 5'-TGA[CG]TCA-3'. Promotes activity of NR5A1 when phosphorylated by HIPK3 leading to increased steroidogenic gene expression upon cAMP signaling pathway stimulation. |
| NQO1 | NAD(P)H dehydrogenase [quinone] 1 | The enzyme apparently serves as a quinone reductase in connection with conjugation reactions of hydroquinons involved in detoxification pathways as well as in biosynthetic processes such as the vitamin K-dependent gamma-carboxylation of glutamate residues in prothrombin synthesis; Belongs to the NAD(P)H dehydrogenase (quinone) family. |
| NOS3 | Nitric-oxide synthase, endothelial | Nitric oxide synthase, endothelial; Produces nitric oxide (NO) which is implicated in vascular smooth muscle relaxation through a cGMP-mediated signal transduction pathway. NO mediates vascular endothelial growth factor (VEGF)-induced angiogenesis in coronary vessels and promotes blood clotting through the activation of platelets. |
| LEPR | Leptin receptor | Receptor for hormone LEP/leptin (Probable). On ligand binding, mediates LEP central and peripheral effects through the activation of different signaling pathways such as JAK2/STAT3 and MAPK cascade/FOS. |
| NAMPT | Nicotinamide phosphoribosyltransferase | Catalyzes the condensation of nicotinamide with 5- phosphoribosyl-1-pyrophosphate to yield nicotinamide mononucleotide, an intermediate in the biosynthesis of NAD. It is the rate limiting component in the mammalian NAD biosynthesis pathway. The secreted form behaves both as a cytokine with immunomodulating properties and an adipokine with anti-diabetic properties, it has no enzymatic activity, partly because of lack of activation by ATP. |
| CDKN2A | Cyclin-dependent kinase inhibitor 2A | Acts as a negative regulator of the proliferation of normal cells by interacting strongly with CDK4 and CDK6. This inhibits their ability to interact with cyclins D and to phosphorylate the retinoblastoma protein. |
| CDKN1A | Cyclin-dependent kinase inhibitor 1 | be involved in p53/TP53 mediated inhibition of cellular proliferation in response to DNA damage. Binds to and inhibits cyclin-dependent kinase activity, preventing phosphorylation of critical cyclin-dependent kinase substrates and blocking cell cycle progression. Functions in the nuclear localization and assembly of cyclin D-CDK4 complex and promotes its kinase activity towards RB1. |
| SNAI1 | Zinc finger protein SNAI1 | Involved in induction of the epithelial to mesenchymal transition (EMT), formation and maintenance of embryonic mesoderm, growth arrest, survival and cell migration. Binds to 3 E-boxes of the E-cadherin/CDH1 gene promoter and to the promoters of CLDN7 and KRT8 and, in association with histone demethylase KDM1A which it recruits to the promoters, causes a decrease in dimethylated H3K4 levels and represses transcription. |

Supplementary Table 9. The correlation coefficient between ferroptosis and autophagy-related hub genes and immune cells.

|  | **IL6** | **PTGS2** | **JUN** | **NQO1** | **NOS3** |
| --- | --- | --- | --- | --- | --- |
| B cell | -0.458766121 | -0.368967693 | -0.469761847 | 0.566279886 | -0.397067881 |
| T cell CD4+ central memory | 0.240573961 | 0.203327739 | 0.510456425 | -0.409708446 | 0.422836213 |
| T cell CD4+ effector memory | 0.399945233 | 0.455925126 | 0.722140615 | -0.587166874 | 0.543004959 |
| T cell CD8+ | -0.015921691 | 0.131047767 | 0.146357085 | -0.388244318 | -0.091855911 |
| Common lymphoid progenitor | -0.191086691 | -0.236874237 | -0.263736264 | 0.475579976 | -0.352258852 |
| Myeloid dendritic cell | 0.288810872 | 0.570905212 | 0.301938639 | -0.341627236 | 0.206686005 |
| Endothelial cell | 0.42613365 | 0.402940599 | 0.361193108 | -0.433555426 | 0.296561807 |
| Eosinophil | 0.30321137 | 0.516502769 | 0.485506465 | -0.502999428 | 0.429651739 |
|  | **LEPR** | **NAMPT** | **CDKN2A** | **CDKN1A** | **SNAI1** |
| B cell | -0.381185166 | 0.558338528 | -0.288332369 | -0.113622501 | -0.389737397 |
| T cell CD4+ central memory | 0.028697909 | -0.52175241 | 0.123645246 | 0.396275382 | 0.371241036 |
| T cell CD4+ effector memory | 0.289851444 | -0.522478998 | 0.597740854 | 0.728360603 | 0.692906671 |
| T cell CD8+ | 0.18309945 | -0.46907752 | 0.161054031 | 0.017146437 | -0.079608456 |
| Common lymphoid progenitor | -0.181318681 | 0.412698413 | -0.114774115 | -0.037240537 | -0.188644689 |
| Myeloid dendritic cell | 0.624637467 | -0.346511987 | 0.537322552 | 0.168523891 | 0.353533816 |
| Endothelial cell | 0.552922326 | -0.248629502 | 0.065559023 | 0.179050351 | 0.342020186 |
| Eosinophil | 0.365510872 | -0.515275192 | 0.348017908 | 0.233853303 | 0.421058704 |

Red labelled, p<0.05
