# Supplementary figures and images for: Ferroptosis and Autophagy-Related Genes in the Pathogenesis of Ischemic Cardiomyopathy
Source: Front Cardiovasc Med. 2022 Jun 30;9:906753. doi: 10.3389/fcvm.2022.906753 (PMC9279674; doi:10.3389/fcvm.2022.906753)

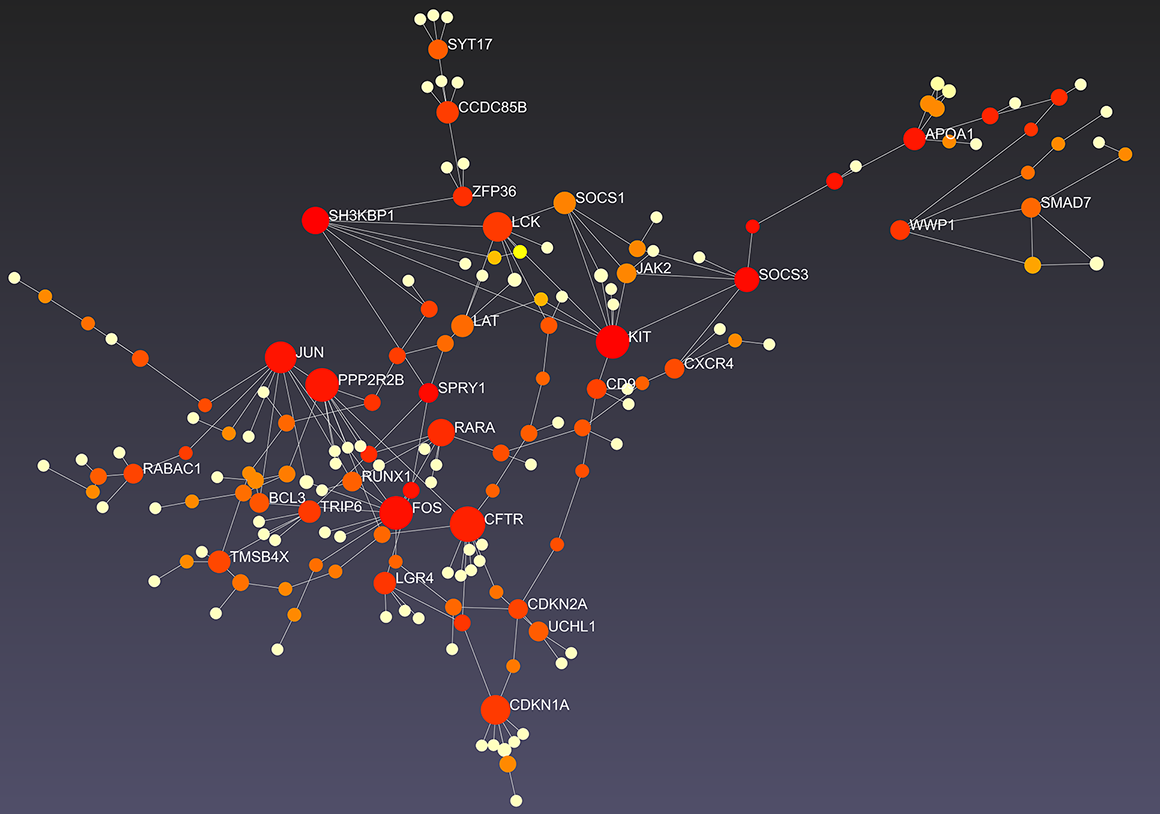

Supplement: Supplementary Figure 1 — The PPI network of the DEGs in GSE116250. [file Image_1.TIF]

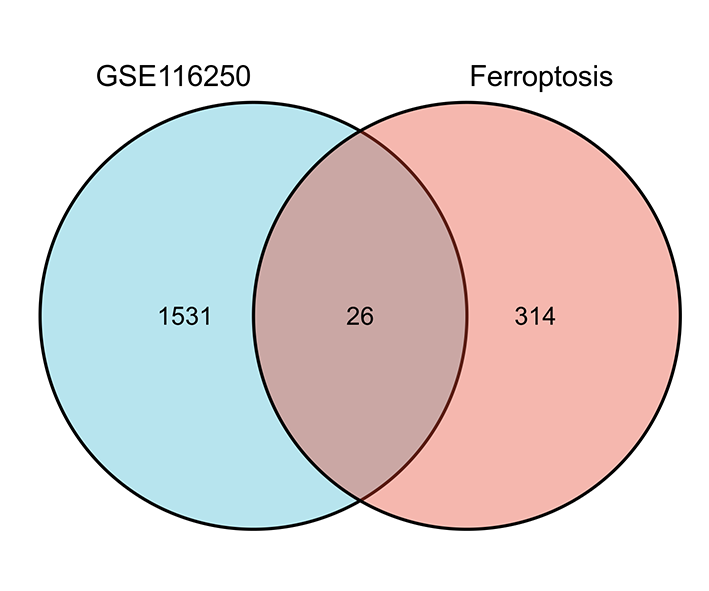

Supplement: Supplementary Figure 2 — A Venn diagram of DEGs and ferroptosis genes. [file Image_2.TIF]

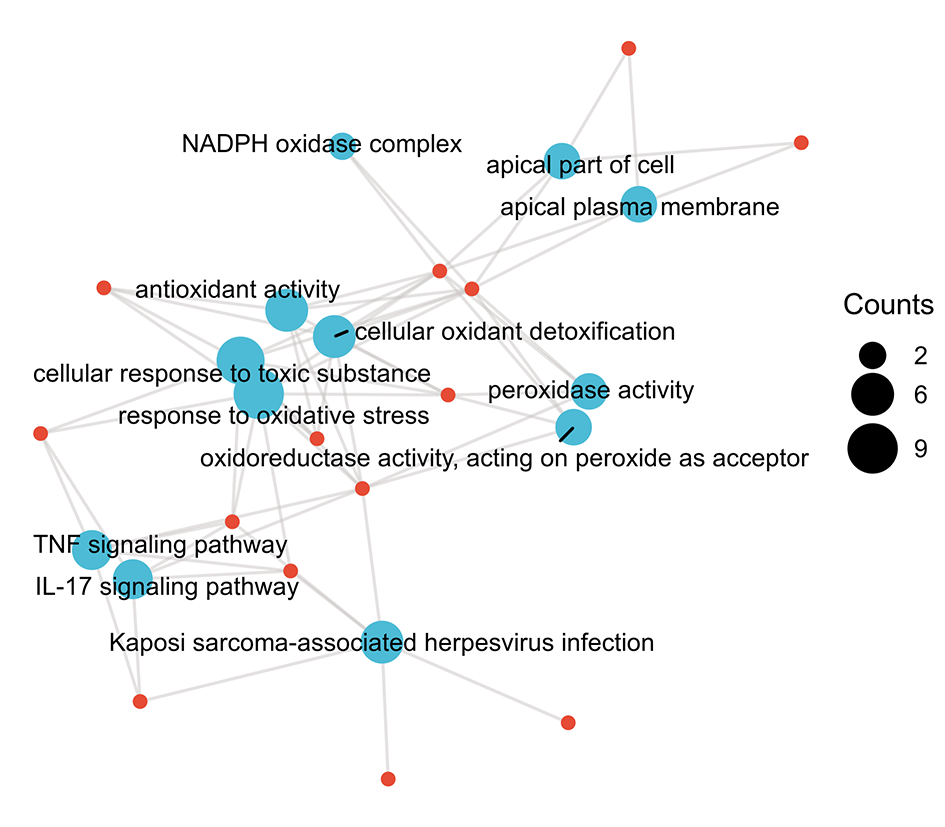

Supplement: Supplementary Figure 3 — The network of the enriched pathways of ferroptosis-related genes. [file Image_3.TIF]

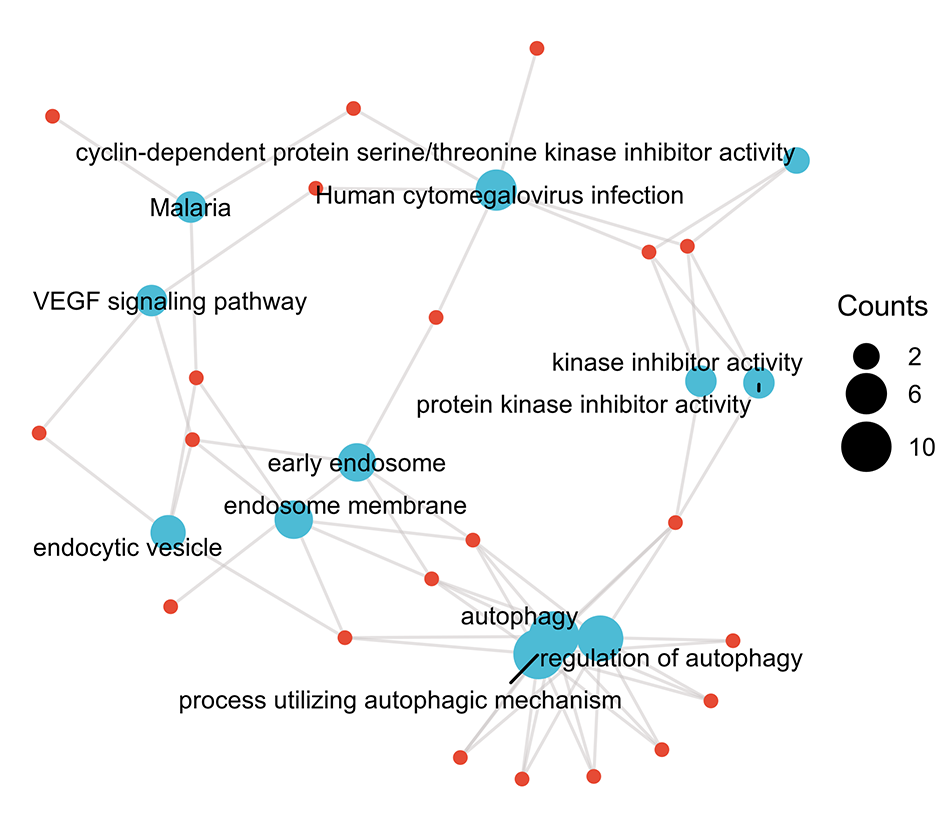

Supplement: Supplementary Figure 4 — The network of the enriched pathways of autophagy-related genes. [file Image_4.TIF]

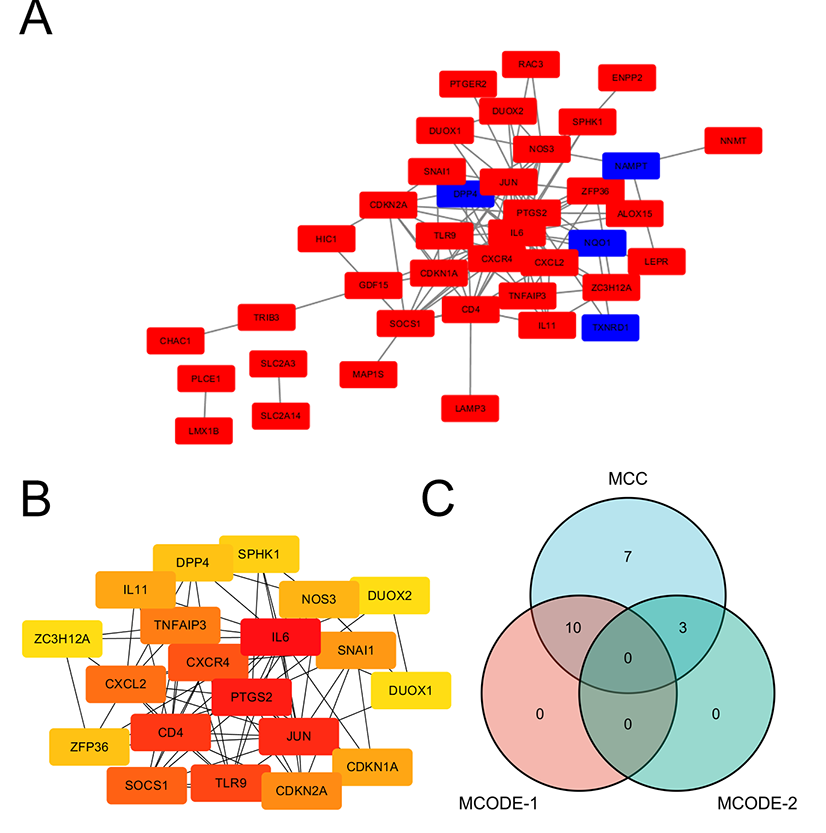

Supplement: Supplementary Figure 5 — The PPI analysis of ferroptosis-related and autophagy-related DEGs together. (A) The PPI analysis of ferroptosis-related and autophagy-related DEGs together. Red, upregulated genes; blue, downregulated genes. (B) The top 20 of the DEGs mentioned above using MCC. (C) A Venn diagram of the DEGs mentioned above using MCC and MCODE analysis module. [file Image_5.TIF]
